# SARS-CoV-2 spike IgG titres up to 137 days following Comirnaty mRNA COVID-19 vaccination, Israel, February to May 2021

**DOI:** 10.2807/1560-7917.ES.2022.27.40.2100703

**Published:** 2022-10-06

**Authors:** Tal Patalon, Shay Ben Moshe, Asaf Peretz, Ami Neuberger, Licita Schreiber, Rachel Lazar, Lia Supino-Rosin, Galit Perez, Miri Mizrahi-Reuveni, Sivan Gazit

**Affiliations:** 1 Kahn Sagol Maccabi (KSM) Research & Innovation Center, Maccabi Healthcare Services, Tel Aviv, Israel; 2 Department of Computer Science, Ben-Gurion University, Beer Sheva, Israel; 3 Internal Medicine COVID-19 Ward, Samson Assuta Ashdod University Hospital, Ashdod Israel; 4 Infectious Diseases Institute, Rambam Healthcare Campus, Haifa, Israel; 5 The Ruth and Bruce Rappaport Faculty of Medicine, Technion Institute of Technology, Haifa, Israel; 6 Central Laboratory, Maccabi Healthcare Services, Rehovot, Israel; 7 Health Division, Maccabi Healthcare Services, Tel Aviv, Israel

**Keywords:** COVID-19, coronavirus, vaccination, serology, SARS-CoV-2, IgG antibodies

## Abstract

**Background:**

Data regarding the long-term protection afforded by vaccination for the SARS-CoV-2 infection are essential for allocation of scarce vaccination resources worldwide.

**Methods:**

We conducted a retrospective cohort study aimed at studying the kinetics of IgG antibodies against SARS-CoV-2 in COVID-19-naïve patients fully vaccinated with two doses of Comirnaty mRNA COVID-19 vaccine. Geometric mean concentrations (GMCs) of antibody levels were reported. Linear models were used to assess antibody levels after full vaccination and their decline over time.

**Results:**

The study included 4,740 patients and 5,719 serological tests. Unadjusted GMCs peaked 28–41 days after the first dose at 10,174 AU/mL (95% CI: 9,211–11,237) and gradually decreased but remained well above the positivity cut-off. After adjusting for baseline characteristics and repeated measurements, the antibodies half-life time was 34.1 days (95% CI: 33.1–35.2), and females aged 16–39 years with no comorbidities had antibody levels of 20,613 AU/mL (95% CI: 18,526–22,934) on day 28 post-first-dose. Antibody levels were lower among males (0.736 of the level measured in females; 95% CI: 0.672–0.806), people aged 40–59 (0.729; 95% CI: 0.649–0.818) and ≥ 60 years (0.452; 95% CI: 0.398–0.513), and patients having haematological (0.241; 95% CI: 0.190–0.306) or solid malignancies (0.757; 95% CI: 0.650–0.881), chronic kidney disease with glomerular filtration rate (GFR) ≥ 30 (0.434; 95% CI: 0.354–0.532) or with GFR < 30 mL/min (0.176; 95% CI: 0.109–0.287), and immunosuppression (0.273; 95% CI: 0.235–0.317). Body mass index, cardiovascular disease, congestive heart failure, chronic obstructive pulmonary disease, diabetes and inflammatory bowel diseases were not associated with antibody levels.

**Conclusions:**

Vaccination with two doses resulted in persistently high levels of antibodies (≥ cut-off of 50 AU/mL) up to 137 days post-first-dose. Risk factors for lower antibody levels were identified.

**Key public health message**

**What did you want to address in this study?**

We attempted to understand the protection afforded by vaccination with the Comirnaty mRNA COVID-19 vaccine by examining the levels of IgG antibodies against SARS-CoV-2 in people who had not had COVID-19.

**What have we learnt from this study?**

Up to May 2021, shortly after COVID-19 vaccines had become available, we observed persistently high levels of antibodies up to ca 20 weeks after vaccination. Antibody levels were lower in older people, males, people with haematological and solid malignancies, people with chronic kidney disease and those immunocompromised.

**What are the implications of your findings for public health?**

When vaccine availability, vaccine costs and vaccine hesitancy are considered, different vaccination schedules for different populations might be considered, including scheduling of additional vaccine doses. Such schedules would ideally depend on factors that affect the duration of protection, further insights on immune status, and on the risk for severe COVID-19 in each specific population.

## Introduction

Prioritisation of coronavirus disease (COVID-19) vaccination has important medical, economic, and social implications worldwide. While in some countries vaccination programmes hardly began by the first half of 2021 and infection rates were high, other countries had already achieved considerable success in curbing the pandemic. In both scenarios, data regarding the long-term persistence of protection afforded by vaccination against the severe acute respiratory syndrome coronavirus 2 (SARS-CoV-2) infection are essential for the efficient allocation of scarce vaccination resources worldwide. The very fact that the optimal approach to revaccination of people who were fully immunised against disease in the past is currently not well-defined, creates a major problem.

SARS-CoV-2 spike IgG titres are readily measurable and seropositivity is associated with protection against COVID-19 [1]. However, few observations about long-term antibody persistence and SARS-CoV-2 immunity are available (as at summer 2021). Most cohorts include previously infected, rather than vaccinated, individuals [2,3]. They show persistence of protective antibody levels for several months after naturally occurring infection, and a gradual decline of antibody levels, with lower antibody levels measured in older patients with comorbid conditions [4-7].

The aim of this study was to use a large patient cohort from Maccabi Healthcare Services (MHS), the second largest Health Maintenance Organisation in Israel, to describe antibody persistence over time in vaccinated SARS-CoV-2-naïve patients. In addition, we also attempted to identify factors that are associated with antibody levels among vaccinated individuals.

## Methods

### Data source

This study was conducted with the use of data from the central computerised database of MHS, which includes more than 2.5 million members (25% of the population) and provides a representative sample of the Israeli population.

### Study design and participants

This retrospective cohort study aimed to study the kinetics of IgG antibodies against SARS-CoV-2 in MHS members who had previously received the Comirnaty mRNA COVID-19 vaccine (BNT162b2, BioNTech-Pfizer, Mainz, Germany/New York, United States), by observing IgG serology test results. Serological tests were not performed routinely but were widely and freely available to vaccinated persons in the community and performed if requested by attending physicians or by the vaccinees themselves.

To assess antibody levels in fully vaccinated individuals, we included only people who had been vaccinated twice within the 21-to-27-day interval set by the national guidelines. Most of the Israeli population followed these guidelines – exceptions included proven infection after the first dose or an intercurrent illness that delayed the administration of the second dose. In an attempt to describe antibody responses to vaccination, rather than to actual infection, patients were excluded from the study if they had a positive SARS-CoV-2 PCR assay test result by the end of the study period. The study included data on vaccinations and PCR testing performed before the end of the study period on 20 May 2021. Serological tests were performed between 1 February 2021 and 6 May 2021. This time frame would allow for enough time for PCR tests to be performed after serological testing, thereby enabling us not to include patients tested following a clinical infection or those who were infected immediately following vaccination. Participants included were all aged ≥ 16 years, the minimal age for Comirnaty vaccination in Israel during the study period.

### Study variables

The administration date of the first dose and the number of days between the first and second doses were recorded for each patient. Individual-level clinical and demographic data on the study population were collected at the time of administration of the first vaccine dose. These data included age, sex (binary variable), socioeconomic status (SES), based on a score ranked of 1 to 10 (lowest to highest) [8], body mass index (BMI), data on chronic diseases from MHS automated registries, including cardiovascular diseases [9], congestive heart failure (CHF), chronic kidney disease (CKD) [10], chronic obstructive pulmonary disease (COPD), diabetes [11], immunocompromised conditions and inflammatory bowel diseases (IBD) [12], as well as data on cancer from the National Cancer Registry [13].

BMI was categorised using standard cut-points: underweight (< 18.5 kg/m^2^), normal weight (18.5–24.9 kg/m^2^), overweight (25.0–29.9 kg/m^2^) or obese (≥ 30.0 kg/m^2^) [14].

Serology test results were recorded, along with the number of days between the time of first dose administration and the time that the serology test was performed.

### Antibody measurement

SARS-CoV-2 serology testing was performed with the SARS-CoV-2 IgG II Quant assay by Abbott, measuring IgG antibodies against the receptor-binding domain (RBD) part of the S1 subunit of SARS-CoV-2. Results are reported in arbitrary units (AU)/mL. In accordance with the manufacturer's instructions, values ≥ 50 AU/mL were interpreted as positive. Values < 21 AU/mL were reported as 21 AU/mL, and values > 40,000 AU/mL were reported as 40,000 AU/mL.

### Statistical analyses

Geometric mean concentrations (GMCs) of IgG antibody levels were reported in periods of 14 days from the first dose and were also further stratified by age groups and by comorbidities. GMCs, along with associated 95% confidence intervals (CI), were calculated by applying logarithm to the results, computing their mean and CI using the Student's t-distribution, and exponentiating to express in the original scale.

To assess the persistence of antibodies over time, as well as the effect of comorbidities on antibody levels, two linear models were applied to the tests performed, starting 28 days after the first dose. In both models the outcome was the logarithm of the test result. To express in the original scale, regression parameters were exponentiated, so that parameters other than the intercept should be interpreted as multiplicative factors relative to the intercept. For all models, the reference group which served as a baseline for analysis was that of young (16–39 years old), healthy female individuals [15,16].

The first model, which did not take into account repeated measurements and baseline characteristics, was an ordinary least squares linear regression with days from first dose minus 28 days as the only predictor (so that the intercept reflects the GMC on day 28).

The second model was a linear mixed model, taking into account that several serological-test results could belong to the same individual (i.e. repeated measurements) and grouping such results, with days from first dose minus 28 days, sex, age groups, BMI and comorbidities as predictors.

In both models, the number of days from first dose was treated as a continuous variable, while the other variables were treated as categorical (female individuals aged 16–39 years with normal BMI and no comorbidities were used as the baseline for comparison).

As a sensitivity analysis, the effect of the interaction between days from the first vaccine dose and baseline characteristics on antibody levels was assessed using a similar linear mixed model (taking into account repeated measurements) with interaction terms added as continuous variables.

All analyses were performed using Python 3.8.1, with the statsmodels, pandas, numpy and matplotlib packages.

## Results

During the study period, a total of 5,189 MHS members performed at least one serological test after the administration of the first dose of the vaccine. Among them, 279 did not receive the second dose or received it more than 27 days after the first dose and were therefore excluded from the study. Of the remaining patients, 170 had a positive PCR and were also excluded. The remaining 4,740 patients were included in the study.

Baseline demographic and clinical characteristics of the cohort appear in Table 1. Of the cohort 59.6% were female. Young adults aged 16 to 39 years constituted 32.6% of the patients, 38.2% were middle-aged (40 to 59 years old), and 29.2% were aged ≥ 60 years.

**Table 1:** Baseline characteristics of the study population and the entire MHS population aged ≥ 16 years, Israel, February–May 2021 (n = 1,782,247 individuals)

Variable		Study population		All MHS members ≥ 16 years old	
		Number	Frequency (%)	Number	Frequency (%)
		Total: 4,740	100	Total: 1,782,247	100
Age group in years	16–39	1,545	32.6	774,667	43.5
	40–59	1,810	38.2	608,810	34.2
	≥ 60	1,385	29.2	398,770	22.4
Sex^a^, male		1,914	40.4	856,313	48.0
SES	Low (1–4)	1,192	25.1	358,266	20.1
	Medium (5–6)	1,364	28.8	568,073	31.9
	High (7–10)	2,179	46.0	850,113	47.7
	Missing	5	0.1	5,795	0.3
BMI in kg/m^2^	Underweight (< 18.5)	163	3.4	82,841	4.6
	Normal weight (18.5–24.9)	1,797	37.9	722,924	40.6
	Overweight (25.0–29.9)	1,546	32.6	523,056	29.3
	Obesity (≥ 30.0)	1,098	23.2	339,359	19.0
	Missing	136	2.9	114,067	6.4
Cancer	Haematological	146	3.1	8,114	0.5
	Solid malignancy	417	8.8	107,422	6.0
Cardiovascular disease		239	5.0	86,456	4.9
CHF		25	0.5	9,825	0.6
CKD in mL/min	GFR ≥ 30	231	4.9	71,403	4.0
	GFR < 30	35	0.7	5,472	0.3
COPD		69	1.5	28,046	1.6
Diabetes		377	8.0	146,074	8.2
Immunocompromised		495	10.4	37,041	2.1
IBD		96	2.0	16,532	0.9

BMI: body mass index; GFR: glomerular filtration rate; CHF: congestive heart failure; CKD: chronic kidney disease; COPD: chronic obstructive pulmonary disease; IBD: inflammatory bowel disease; MHS: Maccabi Healthcare Services; SES: socioeconomic status.

^a^ Sex was collected as a binary variable.

Additionally, Table 1 presents demographic and clinical characteristics of the entire MHS population aged ≥ 16 years, including untested, unvaccinated and previously infected individuals. Overall, the study population appeared to be older and seemed to have more comorbidities than the total population, especially immunosuppression conditions, malignancies and higher BMIs.

The mean number of days between the first and second dose of the vaccine was 21.5 (standard deviation (SD): ± 1.2). Patients in the cohort performed 5,719 serological tests during the study period, reflecting a mean of 1.2 (SD:  ± 0.6) tests per patient, up to 137 days after the administration of the first dose.

**Figure 1:**
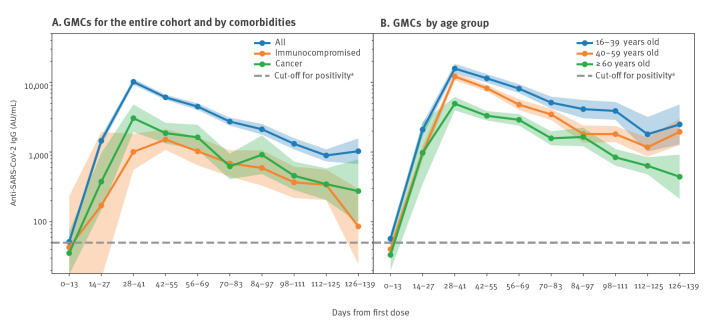
Distribution of GMCs of IgG against SARS-CoV-2 in periods of 14 days from reception of the first vaccine dose for (A) the entire cohort and by comorbidities, as well as (B) by age groups, Israel, February–May 2021 (n = 4,740 patients)

GMC: geometric mean concentration; IgG: immunoglobulin G; SARS-CoV-2: severe acute respiratory syndrome coronavirus 2.

^a^ The cut-off for positivity was 50 AU/mL.

Figure 1 shows IgG antibody levels in relation to the time since administration of the first vaccine dose. The GMCs in each period increment, as well as GMCs further stratified by comorbidities and by age, are also shown. GMCs peaked at 28–41 days after the first dose, with a value of 10,174 AU/mL (95% CI: 9,211–11,237). Although GMCs gradually decreased, they remained well within the positivity range of the test.

Shaded areas around the curves represent 95% confidence intervals.

Somewhat lower antibodies levels were observed on days 28–41 in increasing age groups, with  15,784 AU/mL (95% CI: 13,529–18,416) for 16–39 year-olds, 12,155 AU/mL (95% CI: 10,642–13,883) for 40–59 year-olds and 4,941 AU/mL (95% CI: 3,964–6,160) for ≥ 60 year-olds. This was also the case for patients with cancer (3,060 AU/mL; 95% CI: 1,945–4,813) and immunosuppression (1,006 AU/mL; 95% CI: 555–1,824). Nevertheless, antibody levels remained above the positivity cut-off throughout the study period.

**Figure 2:**
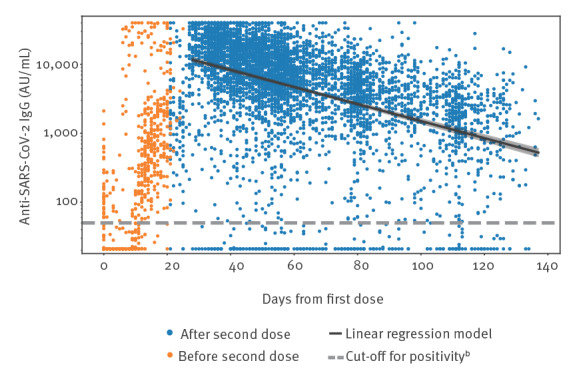
Scatter plot of all serological test results for IgG against SARS-CoV-2, along with the results of a linear regression model, Israel, February–May 2021 (n = 4,740 patients)^a^

IgG: immunoglobulin G; SARS-CoV-2: severe acute respiratory syndrome coronavirus 2.

^a^ For the linear regression model, a total of 4,713 results of tests performed from day 28 post-administration of the first vaccine dose are included. The tests are from 3,763 patients.

^b^ The cut-off for positivity is 50 AU/mL.

A total of 4,713 tests were performed from day 28 onwards on 3,763 patients, with a mean of 1.3 tests per patient (range: 1–5). In Figure 2, a scatter plot of all tests is shown, alongside a linear regression model for the logarithm of all test results from day 28 onwards, with days from the first dose as the predictor. After exponentiating to the original scale, the antibody level on day 28 was 11,649 AU/mL (95% CI: 10,781–12,588), and the decay coefficient was 0.671 for a period of 14 days (95% CI: 0.655–0.688), reflecting a half-life time of 24.4 days (95% CI: 22.9–26.0).

The shaded area surrounding the linear regression line represents the 95% confidence interval.

The effect of age, sex, BMI and comorbidities on antibody levels, as analysed in a linear mixed model taking in account repeated measurements, are shown in Table 2. For females aged 16–39 years with normal BMI and no comorbidities, antibody level on day 28 was 20,613 AU/mL (95% CI: 18,526–22,934). The coefficient of decay over time was 0.752 per 14 days (95% CI: 0.746–0.759), reflecting a half-life time of 34.1 days (95% CI: 33.1–35.2).

**Table 2:** Linear mixed model assessing the effect of days from first dose, age, sex, BMI and comorbidities on SARS-CoV-2 IgG levels found among tests performed on individuals at least 28 days post-reception of the first vaccine dose, Israel, February–May 2021 (n = 3,763 patients)^a^

Variables		Parameter (95% CI)	p value
Reference for the whole table			
**IgG concentration levels in AU/mL, on day 28 post first-dose-vaccination in females aged 16–39 years with normal BMI and no comorbidities (reference group)**		**20,613 (18,526–22,934)**	**< 0.001**
Factors tested for their influence on antibody levels and results			
**Coefficient of decay over 14 days**		**0.752 (0.746–0.759)**	**< 0.001**
Age group in years	16–39	1	NA
	40–59	0.729 (0.649–0.818)	< 0.001
	≥ 60	0.452 (0.398–0.513)	< 0.001
Sex^b^	Female	1	NA
	Male	0.736 (0.672–0.806)	< 0.001
BMI in kg/m^2^	Underweight (< 18.5)	0.950 (0.722–1.250)	0.715
	Normal weight (18.5–24.9)	1	NA
	Overweight (25.0–29.9)	1.042 (0.937–1.159)	0.446
	Obesity (≥ 30.0)	1.106 (0.983–1.244)	0.095
	Missing	0.946 (0.696–1.285)	0.721
Comorbidities^c^	Cancer (haematological)	0.241 (0.190–0.306)	< 0.001
	Cancer (solid malignancies)	0.757 (0.650–0.881)	< 0.001
	Cardiovascular condition	0.866 (0.706–1.062)	0.167
	CHF	0.975 (0.557–1.708)	0.929
	CKD (GFR ≥ 30 mL/min)	0.434 (0.354–0.532)	< 0.001
	CKD (GFR < 30 mL/min)	0.176 (0.109–0.287)	< 0.001
	COPD	0.737 (0.527–1.030)	0.074
	Diabetes	1.022 (0.867–1.204)	0.796
	Immunocompromised	0.273 (0.235–0.317)	< 0.001
	IBD	1.360 (1.010–1.832)	0.043

AU: arbitrary units; BMI: body mass index; CHF: congestive heart failure; CI: confidence interval; CKD: chronic kidney disease; COPD: chronic obstructive pulmonary disease; GFR: glomerular filtration rate; IBD: inflammatory bowel disease; IgG: immunoglobulin G; NA: not applicable.

^a^ 4,713 tests were used in the analysis and these came from 3,763 patients.

^b^ Sex was collected as a binary variable.

^c^ Each comorbidity represents a separate binary variable (i.e. an individuals may have more than one comorbidity; comorbidities are independent of each other).

Parameters were exponentiated to be expressed in the original scale.

Sex was significantly associated with antibody levels, with males having 0.736 (95% CI: 0.672–0.806) times the antibody levels of females. Higher age was also significantly associated with lower antibody titres. Antibody levels in 40–59-year-olds and in people aged ≥ 60 years were respectively 0.729 (95% CI: 0.649–0.818) and 0.452 (95% CI: 0.398–0.513) times those of 16–39-year-olds. Antibody levels were also lower among patients with either haematological (0.241; 95% CI: 0.190–0.306) or solid malignancies (0.757; 95% CI: 0.650–0.881), CKD with glomerular filtration rate (GFR) ≥ 30 mL/min (0.434; 95% CI: 0.354–0.532) or with GFR < 30 mL/min (0.176; 95% CI: 0.109–0.287), and immunosuppression (0.273; 95% CI: 0.235–0.317). In this model, BMI, cardiovascular disease, CHF, COPD, diabetes, and IBD were not associated with statistically significant lower values of antibody levels.

Similar results were obtained when interaction terms between days from first dose and baseline characteristics were added to the linear mixed model, as shown in Table 3.

**Table 3:** Linear mixed model including interaction terms between days from first dose and baseline characteristics**,** Israel, February–May 2021 (n = 3,763 patients)^a^

Variables		Parameter (95% CI)	p value
Reference for the whole table			
**IgG concentration levels in AU/mL, on day 28 post first-dose-vaccination in females aged 16–39 years with normal BMI and no comorbidities (reference group)**		**22,276 (19,756–25,118)**	**< 0.001**
Factors tested for their influence on antibody levels and results			
**Coefficient of decay over 14 days**		**0.726 (0.706–0.746)**	**< 0.001**
Age group in years	16–39	1	NA
	40–59	0.724 (0.636–0.825)	< 0.001
	≥ 60	0.417 (0.362–0.481)	< 0.001
Sex^b^	Female	1	NA
	Male	0.710 (0.642–0.785)	< 0.001
BMI (kg/m^2^)	Underweight	1.088 (0.797–1.485)	0.595
	Normal weight	1	NA
	Overweight	1.064 (0.948–1.193)	0.291
	Obesity	1.155 (1.016–1.314)	0.028
	Missing	0.915 (0.658–1.272)	0.597
Comorbidities^c^	Cancer (haematological)	0.182 (0.142–0.234)	< 0.001
	Cancer (solid malignancy)	0.636 (0.539–0.750)	< 0.001
	Cardiovascular disease	1.038 (0.829–1.299)	0.747
	CHF	0.878 (0.451–1.709)	0.701
	CKD (GFR ≥ 30 mL/min)	0.430 (0.343–0.538)	< 0.001
	CKD (GFR < 30 mL/min)	0.169 (0.098–0.289)	< 0.001
	COPD	0.843 (0.581–1.224)	0.37
	Diabetes	1.111 (0.927–1.331)	0.254
	Immunocompromised	0.242 (0.206–0.284)	< 0.001
	IBD	1.516 (1.100–2.089)	0.011
Interaction			
Age in years × 14 days	16–39	1	NA
	40–59	1.005 (0.976–1.034)	0.757
	≥ 60	1.038 (1.008–1.069)	0.012
Sex^b^ × 14 days	Female	1	NA
	Male	1.013 (0.995–1.031)	0.168
BMI (kg/m^2^) × 14 days	Underweight (< 18.5)	0.941 (0.879–1.008)	0.083
	Normal weight (18.5–24.9)	1	NA
	Overweight (25.0–29.9)	0.990 (0.971–1.009)	0.287
	Obesity (≥ 30.0)	0.982 (0.961–1.004)	0.1
	Missing	1.017 (0.960–1.077)	0.574
Comorbidities^c^ × 14 days	Cancer (haematological)	1.112 (1.080–1.145)	< 0.001
	Cancer (other)	1.073 (1.047–1.100)	< 0.001
	Cardiovascular	0.933 (0.901–0.967)	< 0.001
	CHF	1.035 (0.901–1.190)	0.625
	CKD (GFR ≥ 30 mL/min)	1.001 (0.965–1.038)	0.97
	CKD (GFR < 30 mL/min)	1.004 (0.922–1.094)	0.928
	COPD	0.957 (0.900–1.017)	0.153
	Diabetes ×	0.972 (0.942–1.003)	0.074
	Immunocompromised	1.048 (1.023–1.073)	< 0.001
	IBD	0.958 (0.916–1.003)	0.066

BMI: body mass index; CHF: congestive heart failure; CI: confidence interval; CKD: chronic kidney disease; COPD: chronic obstructive pulmonary disease; IBD: inflammatory bowel disease.

^a^ 4,713 tests were used in the analysis and these came from 3,763 patients.

^b^ Sex was collected as a binary variable.

^c^ Each comorbidity represents a separate binary variable (i.e. an individuals may have more than one comorbidity; comorbidities are independent of each other).

Parameters were exponentiated to be expressed in the original scale.

## Discussion

In this cohort of patients, all of whom were vaccinated for SARS-CoV-2, we observed persistence of high levels of anti-SARS-CoV-2 antibodies for a period of up to 137 days (i.e. 4.5 months). Lower antibody levels were observed among older patients, males, patients with haematological and solid malignancies, patients with CKD and immunocompromised patients, but still above the positivity cut-off.

Younger persons have a stronger immunological response to vaccines in general, and to the Comirnaty mRNA COVID-19 vaccine specifically. This may explain higher efficacy, as well as a greater number of side effects among young adults vaccinated for COVID-19 [17,18]. The lower antibody levels among older individuals, and the increased risk of severe COVID-19 in patients older than 50 years, suggest that in a reality of limited vaccine availability, older patients should be immunised more often. Previous data have shown that antibody levels following infection gradually decline but remain relatively high for at least 6 months. Our data provide similar reassurance with regards to elderly, vaccinated individuals as antibody levels remained high for nearly 5 months and suggest that revaccination would probably not be necessary for probably much longer following two doses of the vaccine.

Female sex is associated with higher antibody levels in this cohort, as well as in studies assessing immune responses to other vaccines such as influenza and the measles, mumps, and rubella (MMR) vaccines [19-21]. Oestrogen has been shown to promote antibody production; a process mediated by cytokines interleukins (IL)-4 and Il-5 [22], but other genetic and epigenetic differences between males and females probably also exist [20,23]. A stronger immune response to vaccines may result in an excess of reported side effects, an observation that has been repeatedly reported for COVID-19 mRNA vaccines and vaccines for various other diseases [24-26]. Whether these sex-dependent immunological differences are clinically meaningful is not known.

Solid and haematological malignancies, CKD and immunosuppression were all previously shown to be associated with lower antibody levels following clinical infection with SARS-CoV-2 [4-7]. Our results are in line with these observations; it seems that people who mount a less robust immunological response to infection will also have lower titre of anti-SARS-CoV-2 antibodies following vaccination. However, the majority of these patients still retained a relatively high level of antibodies several months after receiving the second dose of the Comirnaty mRNA COVID-19 vaccine. This provides some level of reassurance to both patients and clinicians.

Diabetes and IBD were not associated with lower levels of anti-SARS-CoV-2 antibodies. In the community setting, most diabetic patients are not considered immunocompromised. Similarly, many patients with IBD are in remission most of the time, thus leaving very small number of patients with IBD who currently have active disease. IBD patients who are treated with either corticosteroids or other immunomodulating agents would be included in the immunosuppressed group in this analysis. IBD by itself, therefore, is probably not a condition associated with lower antibody levels if in remission.

Our study has several limitations. Though the time frame is relatively long when compared with currently available data, it is still not sufficient for making decisions regarding revaccination in the long term. It seems that antibody levels remain in the positive range for at least 1 year, but whether this observation is well correlated with clinical effectiveness is unknown. A selection bias probably exists as persons were either referred for testing or chose to be tested. As seen in Table 1, the study population was older and had more comorbidities, including being more immunocompromised; hence antibody levels may be lower when compared with the general population. We have tried to address these biases via the use of a multivariate model, considering several comorbidities as well as immunosuppression with in particular, the sensitivity analysis. Interestingly, despite this, it seems that the rate of decay is relatively similar between immunocompromised patients and immunocompetent ones. Some unknown confounders, however, may remain. Lastly, although anti-SARS-CoV-2 antibodies levels are a correlate of the immunological response to vaccination, we did not assess the cellular components of this response.

Decay of anti-SARS-CoV-2 antibodies has been described for patients recovering from COVID-19, and the antibody half-life measured among patients with mild COVID-19 (36 days) is similar to the one calculated for vaccinees in our cohort [27]. In general, antibody decay often decelerates with time, and antibody levels that confer protection from severe COVID-19 are probably much lower than the levels that confer overall protection [28]. All in all, these observations and our results are encouraging as protection from severe COVID-19 is likely to be retained for many months despite a significant decline in antibody levels. Nonetheless, even today, months after the execution of this study, correlation of protection (in the context of antibody level) has yet to be sufficiently understood. When vaccine availability (especially in areas around the world where shortage exists), vaccine costs and vaccine hesitancy are considered, it would perhaps be wiser to apply different vaccination schedules for different populations, including scheduling of additional doses. Such schedules would ideally depend both on factors that affect the duration of protection, further research results on immunity maturation, and on the risk for severe COVID-19 in each specific population.
